# The association between poor glycemic control and PTSD in patients with diabetes mellitus in primary healthcare in Palestine

**DOI:** 10.3389/fmed.2026.1783847

**Published:** 2026-06-01

**Authors:** Muna Ahmead, Hala Taqatqa, Maisa Nabulsi

**Affiliations:** 1 Faculty of Public Health, Al-Quds University, Jerusalem, Palestine; 2 Faculty of Pharmacy, Al-Quds University, Jerusalem, Palestine

**Keywords:** diabetes complications, distress, health and wellbeing, Palestine, PTSD, glycemic control

## Abstract

**Background:**

Diabetes mellitus remains a significant global health burden, particularly in low- and middle-income countries and in war and conflict zones. This study aimed to examine the prevalence of poor glycemic control and its association with post-traumatic stress disorder (PTSD), stress, dietary patterns in individuals with type 1 and type 2 diabetes in primary healthcare settings in southern Palestine from October 1, 2025, to December 1, 2025. It also aimed to assess the factors associated with poor glycemic control in these individuals.

**Method:**

A cross-sectional analytical study design was utilized for a sample of 531 patients with diabetes mellitus. Data were collected using self-reported questionnaires, including the PTSD Checklist for DSM-5 (PCL-5), Food Frequency Questionnaire, and Perceived Stress Scale (PSS-10). For HbA1c test results, the information was obtained from the patients’ records, and the test were conducted within the last 2 months at the latest.

**Results:**

The results showed that 76.3% of participants had poor glycemic control. Participants with type 2 diabetes (AOR: 2.348, *p* < 0.015), those with PTSD (AOR: 5.455, *p* < 0.001), and individuals experiencing moderate to high levels of stress were associated with poor glycemic control (AOR: 3.172, *p* < 0.001). Additionally, individuals who followed an average dietary pattern were less likely to experience poor glycemic control (AOR: 0.054, *p* < 0.001).

**Conclusion:**

The findings revealed a high prevalence of poor glycemic control. Therefore, it is essential that psychological interventions and dietary counseling be made available at primary health care centers to support patients with diabetes mellitus.

## 1 Introduction

The global pandemic of diabetes mellitus (DM) poses a significant and escalating challenge to public health and healthcare systems (1). It affects over 537 million individuals globally, and this number is projected to rise to 643 million by 2030 and 783 million by 2045 (2). The International Diabetes Federation (IDF) estimates that 85 million adults (20–79) are affected by diabetes in the Middle East and North Africa (MENA) region in 2024, and isexpected to increase dramatically by 2050, reaching 163 million (3). The two primary categories of diabetes are type 1 (T1D), caused by an insufficiency in *β*-cell activity, and type 2 (T2D), which results from insulin resistance and insufficient insulin production (4). T2D comprises 90–95% of the total cases of diabetes in the United States and worldwide (5). Diabetes significantly increases the risk of developing kidney failure, cardiovascular disease, neuropathy, and nephropathy. These complications significantly increase the morbidity and mortality rates associated with diabetes (6). Poor glycemic control is a significant risk factor in the development of diabetes complications. One systematic review and meta-analysis included studies from Middle Eastern countries (e.g., Saudi Arabia, Turkey, Iran, Jordan, Iraq, Palestine, Egypt, Bahrain, Kuwait, Oman, Lebanon, the United Arab Emirates, and Yemen) found that approximately 63% of the participants with type 2 diabetes had an HbA1c level above 7.0% (7).

Treatment and management of diabetes focus on maintaining satisfactory blood sugar control. Pharmacological, surgical, and lifestyle therapies are currently employed to treat diabetes and control blood sugar (8). Concerns regarding healthcare costs and adverse effects have increased alongside the growing number of pharmacological treatments (9). However, there are effective and cost-efficient strategies to address these issues through dietary modifications (9, 10). These changes can improve insulin sensitivity and glycemic control, reduce reliance on medications, and provide more affordable options for preventing complications (9, 10). According to Evert et al. (11), nutritional therapy remains an important aspect of diabetes management and prevention. Several trials have demonstrated that increasing dietary fiber and modifying macronutrient composition can enhance glycemic management and reduce arterial stiffness (12). Additionally, supplements such as vitamins, selenium, arginine, glutamine, zinc, chromium, magnesium, and selenium may support diabetes control (13). Studies have shown that a low-glycemic-index diet, which is rich in fiber and follows Mediterranean principles, can improve glucose uptake (14). Among these strategies, low-carbohydrate diets (LCDs) have garnered significant attention for their potential effectiveness in managing type 2 diabetes (15).

Individuals often experience stress, limited access to nutritious food, painful separation from family and community, displacement from their homes, and interruptions to essential health and social services in conflict and war zones. These events can lead to increased occurrences of hyperglycemia and hypoglycemia, adversely affecting diabetes management (16). One study indicated that individuals with diabetes had a significantly higher average HbA1c during wartime, rising from 7.7 to 9.4, compared to those without diabetes or with lower risk (17). Additionally, violence and conflict result in severe psychological effects, including heightened levels of anxiety, depression, stress, and post-traumatic stress disorder (PTSD) (18, 19).

Stress is highly prevalent among populations living in war and conflict zones, where ongoing insecurity, displacement, and loss significantly impact mental health (18, 20). The bidirectional relationship between diabetes and stress is well documented. Chronic stress is consistently associated with an increased risk of developing type 2 diabetes (21–24). Additionally, individuals with diabetes report higher stress levels due to the burden of the illness and the demands of self-management, which include blood glucose monitoring, and strict medication adherence (25–27). Chronic stress can impair glycemic regulation by activating the hypothalamic–pituitary–adrenal axis and releasing cortisol and catecholamines, which both contribute to insulin resistance and hyperglycemia (28). Furthermore, stress may indirectly affect glycemic control by reducing adherence to treatment regimens, physical exercise, and healthy eating habits, all of which are essential components of diabetes self-management (29–31). Egede et al. (32) found an association between stress and poor glycemic control, while another study indicated that elevated stress levels decreased treatment adherence and worsened glycemic control (33). Conversely, other studies have failed to show significant correlations between glycemic control and the frequency of daily stress (34).

Furthermore, PTSD is a condition commonly found in populations affected by conflict, as they endure a significant traumatic burden from violence, displacement, and loss. A study in Jordan found that 69.8% of Syrian refugees with diabetes mellitus exhibited symptoms of PTSD (35). Research shows that 90% of individuals with diabetes report having experienced at least one traumatic event (36). Furthermore, 30% of individuals with type 1 diabetes display diabetes-related posttraumatic stress symptoms, while between 30 and 50% of those with type 2 diabetes show similar symptoms (37, 38). The relationship between PTSD and diabetes mellitus is bidirectional and operates through well-documented mechanisms (36, 39–43). Trauma can lead to chronic stress, which results in dysregulation of the hypothalamic–pituitary–adrenal (HPA) axis, peripheral immune inflammation, and the development of diabetes mellitus (39, 40). On the other hand, the experience of diabetes mellitus—both during diagnosis and treatment—can be perceived as aversive, potentially triggering PTSD (43, 44). This association between PTSD and diabetes has significant implications for glycemic control. Studies indicate that individuals with PTSD who are diagnosed with diabetes and have a history of trauma face a heightened risk of poor glycemic control (38, 42). These comorbid mental disorders in adults with diabetes are linked to poor treatment adherence, elevated HbA1c levels, increased emergency admissions, and more frequent hospitalizations (45).

In Palestine, 15.5% of the population had type 1 or type 2 diabetes in 2021 (46). In 2024, the West Bank had 4,439 newly diagnosed cases of diabetes mellitus. The disease affected 147.9 individuals per 100,000 populations, with 2,181 cases among men and 2,258 cases among women. The total number of fatalities resulting from diabetes-related complications was 1,309, resulting in a mortality rate of 43.6 per 100,000 individuals. Furthermore, diabetes mellitus ranked as the third leading cause of mortality in the West Bank, accounting for 14.5% of fatalities in 2024 (47). Conflicts, political instability, occupation, and economic challenges hinder the effective functioning of the healthcare system in Palestine. Israel’s armed forces have governed the West Bank and Gaza Strip since 1967. Over the past 13 years, four wars have erupted in the Gaza Strip (48). On October 7, 2023, a new conflict between Israel and Palestine began in Gaza. Thousands of Palestinians have been killed or injured as a result (49). In the West Bank, Palestinians are experiencing significant psychological trauma due to border closures, military occupation, and other adverse effects, including a collapsing economy, high unemployment, deteriorating living conditions, and a weakened healthcare system (50–52). In addition, these conditions lead to numerous challenges, including insufficient food availability, inadequate nutritional intake, limited access to healthcare facilities, and a scarcity of medications and supplies (53, 54). Seligman et al. revealed that 46% of individuals with diabetes experienced dietary insecurity. Participants who were food-insecure were substantially more likely to report difficulty affording a diabetic diet and to have poor glycemic control than those who were food-secure (55). Additionally, anxiety, depression, and post-traumatic stress disorder (PTSD) are prevalent in Palestinian society, particularly during and following the 2023 Gaza war (50–52). The interplay between PTSD, stress, and dietary behavior in influencing glycemic outcomes has not been previously investigated in a Palestinian clinical population, highlighting a significant gap in the literature that our study directly addresses. For instance, a study conducted in the northern West Bank indicated that 76% of participants had poor glycemic control (56), while another study in Gaza found a rate of 80% (57). However, these two studies focused solely on type 2 diabetes and examined the prevalence of poor glycemic control without exploring its association with PTSD, dietary patterns, or stress. Consequently, our research enhances the understanding of non-communicable diseases (NCDs), such as diabetes, in the context of conflict. This study aimed to examine the prevalence of poor glycemic control and its association with post-traumatic stress disorder (PTSD), stress, and dietary patterns in individuals with type 1 and type 2 diabetes in primary healthcare settings in southern Palestine from October 1, 2025, to December 1, 2025. It also aimed to assess the factors associated with poor glycemic control in these individuals.

## 2 Methods

### 2.1 Study design and participants

The study was an analytic cross-sectional survey conducted from October 1, 2025, to December 1, 2025. It aimed to investigate the prevalence of poor glycemic control and its association with stress, PTSD, dietary patterns among persons with diabetes mellitus primary care in Palestine, specifically in the Bethlehem and Hebron governorates.

The Palestinian Ministry of Health is the principal supplier of health services in the Palestinian territories, managing over 64% of primary health care centers. These health centers do routine medical assessments, dispense antidiabetic drugs, and monitor glycemic levels in persons with diabetes (58). Moreover, these primary healthcare centers provide vital services for the management of diabetes and its associated complications. Family medicine physicians provide outpatient treatment for diabetes patients, including follow-up appointments, prescription renewals, assessments, blood glucose monitoring—such as HbA1c and fasting blood sugar tests—and referrals to secondary or tertiary healthcare services.

Participants attended eleven governmental primary healthcare centers in these regions, where they regularly visited to receive medications and monitor their blood sugar levels.

Using a significance level of 0.05, a 95% confidence level, a 4% margin of error, and a prevalence rate of 76% (56), the study used Survey Monkey to compute the sample size of 436 participants as follows:


$$\text{Sample size}=\frac{\frac{z^{2}\times p(1-p)}{e^{2}}}{1+(\frac{z^{2}\times p(1-p)}{e^{2}N})}$$

- N = population.
- e = margin of error (percentage in decimal form).
- z = z-score* (how many standard deviations data is from the mean).
*95% confidence level is a 1.96 z-score.

After accounting for an expected 17% non-response rate, the study required a sample size of 526 participants. However, a total of 531 participants were recruited. The study included both type 1 and type 2 diabetes patients, encompassing male and female individuals aged 18 years and older. Data were collected through self-administered surveys by the researchers, which included all persons with diabetes who visited the 11 governmental health centers in the Hebron and Bethlehem governorates during the study period (October 1, 2025 to December 1, 2025).

### 2.2 Tools and measures

Participants in the study completed a self-administered questionnaire consisting of four sections. In the first section, participants were queried regarding their age, gender, family income per month, place of residence and governorates, including Hebron and Bethlehem; marital status; level of education; current living arrangements; and employment status. Additionally, they were asked about their medical history such as: When were you first diagnosed with diabetes? What specific type of diabetes were you diagnosed with? What treatments are you using for your diabetes mellitus? Have you been diagnosed with diabetes-related complications such as ocular issues, renal problems, myocardial infarction, cerebrovascular events, or sexual dysfunction? Have you ever received a diagnosis of a psychological disorder? For HbA1c test results, the information was obtained from the patients’ records, and the test should be conducted within the last 2 months at the latest. The value < 7 was considered good glycemic control, and the value ≥ 7 is considered poor glycemic control. The second section included the PCL-5, a 20-item scale that corresponds with the DSM-5 criteria for PTSD symptoms and assesses PTSD in the past month (59). Respondents rate each item on a scale from 0 (“not at all”) to 4 (“extremely”) to indicate how much that specific symptom has impacted them in the past month. The total of the individual ratings for all 20 items yields the overall symptom severity score, which ranges from 0 to 80. A PCL-5 cutoff score more than 33 suggests probable PTSD in a sample as used by other studies (51, 60, 61). A systematic review of the PCL-5 demonstrated that internal consistency for the total score ranged between 0.83 and 0.97, and test–retest reliability ranged between 0.58 and 0.91 (62). Also, Blevins et al. found that PCL-5 scores exhibited strong internal consistency (*α* = 0.94), test–retest reliability (*r* = 0.82), and convergent (*r*s = 0.74 to 0.85) and discriminant (*r*s = 0.31 to 0.60) validity (61). In the current study, Cronbach’s alpha, was 0.87.

The third section included the 15-item Food Frequency Questionnaire (FFQ) (63) which is a nutritional assessment tool commonly used to evaluate an individual’s typical food consumption over a designated time frame (63). Our study utilized the scoring and classification system for dietary pattern categories established by Persson et al. (64). The response options were coded as dichotomous variables, where each question could be answered with either “meets” or “does not meet” the dietary recommendations. Each guideline met earned one point, resulting in a healthy dietary index that ranges from 0 to 15 points. This index identified three dietary patterns: good (meets nine or more guidelines), average (meets six to eight recommendations), and poor (meets five or fewer recommendations) (64). The reliability of the index was supported by a Cronbach’s alpha of 0.70.

The fourth section included the Perceived Stress Scale (PSS-10) (65). It is a self-report measure consisting of ten items designed to evaluate respondents’ perceptions of their lives as unpredictable, uncontrolled, and overwhelming. Each item on the PSS-10 is scored using a 5-point Likert scale that ranges from 0 (never) to 4 (very often). The PSS-10 includes six positively worded items (items 1, 2, 3, 6, 9, and 10, referred to as the positive factor) and four negatively worded items (items 4, 5, 7, and 8, referred to as the negative factor). The negatively worded items were recoded for analysis during the study. Total scores can range from 0 to 40, with higher scores indicating a greater level of perceived stress. Scores ranging from 0 to 13 indicate low stress, those from 14 to 26 indicate moderate stress, and scores between 27 and 40 indicate high perceived stress (66, 67). Cronbach’s alpha, was 0.87.

A committee of six mental health specialists evaluated the scale’s content to ensure cultural appropriateness but did not make any modifications. After the study team’s translation, a qualified translator converted the scale from Arabic to English. We assessed linguistic clarity with 25 individuals diagnosed with diabetes mellitus during the pilot stage, using both the original English questionnaire and the back-translated version to verify its accuracy.

### 2.3 Ethical approval and consent to participate

All procedures used in the study complied with the Declaration of Helsinki. The Al-Quds University Research Ethical Committee approved the study (REF: 27/25). At the start of the survey, written information on the objective and process of data collection was provided. Participants with diabetes mellitus gave written informed consent to participate in this study before filling out the questionnaire.

### 2.4 Statistical analysis

The data were analyzed using SPSS version 25 (IBM Corp., Chicago, IL, USA). The study focused on one primary outcome: the categorization of glycemic control status as either good (HbA1c < 7%) or poor (HbA1c ≥ 7%). Descriptive analysis was presented in terms of frequencies and percentages. Univariate analysis was performed using a chi-square test, with a statistical significance threshold set at *p* < 0.05. Subsequently, binary stepwise logistic regression was conducted, utilizing a *p*-value of less than 0.05 for variable entry, and a p-value greater than 0.10 for variable removal. To ensure comprehensive analysis, no potentially relevant variables were excluded. All study variables, including sociodemographic factors, were included in the initial model, regardless of their significance in the univariate analysis. Results are reported as adjusted odds ratios (AOR) with 95% confidence intervals.

## 3 Results

### 3.1 Sociodemographic characteristics of the participants

The study recruited 531 patients with diabetes mellitus. The findings presented in Table 1 reveal that 54.8% of the participants were female, 46.7% were aged 41 years or older, and 54.4% were married. Additionally, 62.7% of the participants lived in cities, 71.4% had an educational level of 12 years or less, and 69.5% reported a monthly income ranging from $300 to $1,560.

**Table 1:** The association between glycemic control, dietary pattern, PTSD, perceived stress, sociodemographic and medical and psychological history variables of adults with type 1 and type 2 diabetes mellitus from Southern Governorates of Palestine seeking care at primary healthcare centers in October–November 2025 (*N* = 531).

Characteristics		Total		Glycemic control				*p* value
				Poor control		Good control		
		F	%	F	%	F	%	
Age (years)	18–30	140	26.4%	105	25.9%	35	27.8%	0.153
	31–40	143	26.9%	101	24.9%	42	33.3%	
	41–50	125	23.5%	98	24.2%	27	21.4%	
	≥ 51	123	23.2%	101	24.9%	22	17.5%	
What is your gender?	Male	240	45.2%	184	45.4%	56	44.4%	0.846
	Female	291	54.8%	221	54.6%	70	55.6%	
Governorates	Bethlehem	239	45.0%	178	44.0%	61	48.4%	0.379
	Hebron	292	55.0%	227	56.0%	65	51.6%	
Marital status	Single	125	23.6%	80	19.8%	45	35.7%	0.001^*^
	Married	289	54.4%	232	57.3%	57	45.2%	
	Others (divorced, widowed, separated)	117	22.0%	93	23.0%	24	19.0%	
Place of residence	City	333	62.7%	244	60.2%	89	70.6%	0.035*
	Village	198	37.3%	161	39.8%	37	29.4%	
Educational level (years)	≤ 12	379	71.4%	291	71.9%	88	69.8%	0.663
	> 12	152	28.6%	114	28.1%	38	30.2%	
Work status	Employees	186	35.0%	139	34.3%	47	37.3%	0.745
	Unemployed	131	24.7%	105	25.9%	26	20.6%	
	Students	32	6.0%	24	5.9%	8	6.3%	
	Retired	39	7.3%	31	7.7%	8	6.3%	
	Worker (daily paid)	143	27%	106	26.2%	37	29.4%	
Current living status	Live alone	52	9.8%	39	9.6%	13	10.3%	0.331
	Live with nuclear family.	307	57.8%	241	59.5%	66	52.4%	
	Live with friends or roommates.	28	5.3%	18	4.4%	10	7.9%	
	Live with extended family.	144	27.1%	107	26.4%	37	29.4%	
Family monthly income ($)	< 300	70	13.2%	54	13.3%	16	12.7%	0.580
	300–1,000	215	40.5%	161	39.8%	54	42.9%	
	1,001–1,560	154	29.0%	123	30.4%	31	24.6%	
	>1,560	92	17.3%	67	16.5%	25	19.8%	
When were you diagnosed with diabetes for the first time? (years)	< 1	44	8.3%	28	6.9%	16	12.7%	0.202
	1–5	227	42.7%	173	42.7%	54	42.9%	
	6–10	198	37.3%	156	38.5%	42	33.3%	
	> 10	62	11.7%	48	11.9%	14	11.1%	
What type of diabetes were you diagnosed with?	Type 1	200	37.7%	147	36.3%	53	42.1%	0.243
	Type 2	331	62.3%	258	63.7%	73	57.9%	
What treatments are you using for your diabetes mellitus?	Insulin	152	28.6%	44	34.9%	108	26.7%	0.292
	oral drugs	199	37.5%	44	34.9%	155	38.3%	
	Insulin + Oral drugs	129	24.3%	29	23.0%	100	24.7%	
	Herbal/Diet	51	9.6%	9	7.1%	42	10.4%	
Have you received a diagnosis for any complications related to diabetes?	Yes	210	39.5%	168	41.5%	42	33.3%	0.102
	No	321	60.5%	237	58.5%	84	66.7%	
Have you received a diagnosis for any psychological disorders?	Yes	73	13.7%	53	13.1%	20	15.9%	0.428
	No	458	86.3%	352	86.9%	106	84.1%	
PTSD	≤33	389	73.3%	269	66.4%	120	95.2%	<0.001*
	>33	142	26.7%	136	33.6%	6	4.8%	
Perceived stress	Low	244	46.0%	147	36.3%	97	77.0%	<0.001*
	Moderate	229	43.1%	200	49.4%	29	23.0%	
	High	58	10.9%	58	14.3%	0	0.0%	
Dietary pattern	Poor	201	37.9	196	48.4%	5	4.0%	<0.001*
	Average	169	31.8	112	27.7%	57	45.2%	
	Good	161	30.3	97	24.0%	64	50.8%	

PTSD, Post-Traumatic Stress Disorder Score. PTSD, severity was measured using the PTSD Checklist (PCL-5); a score of >33 indicates a high risk of having PTSD symptoms. *p < 0.05.

### 3.2 Prevalence of PTSD and perceived stress

**Figure 1:**
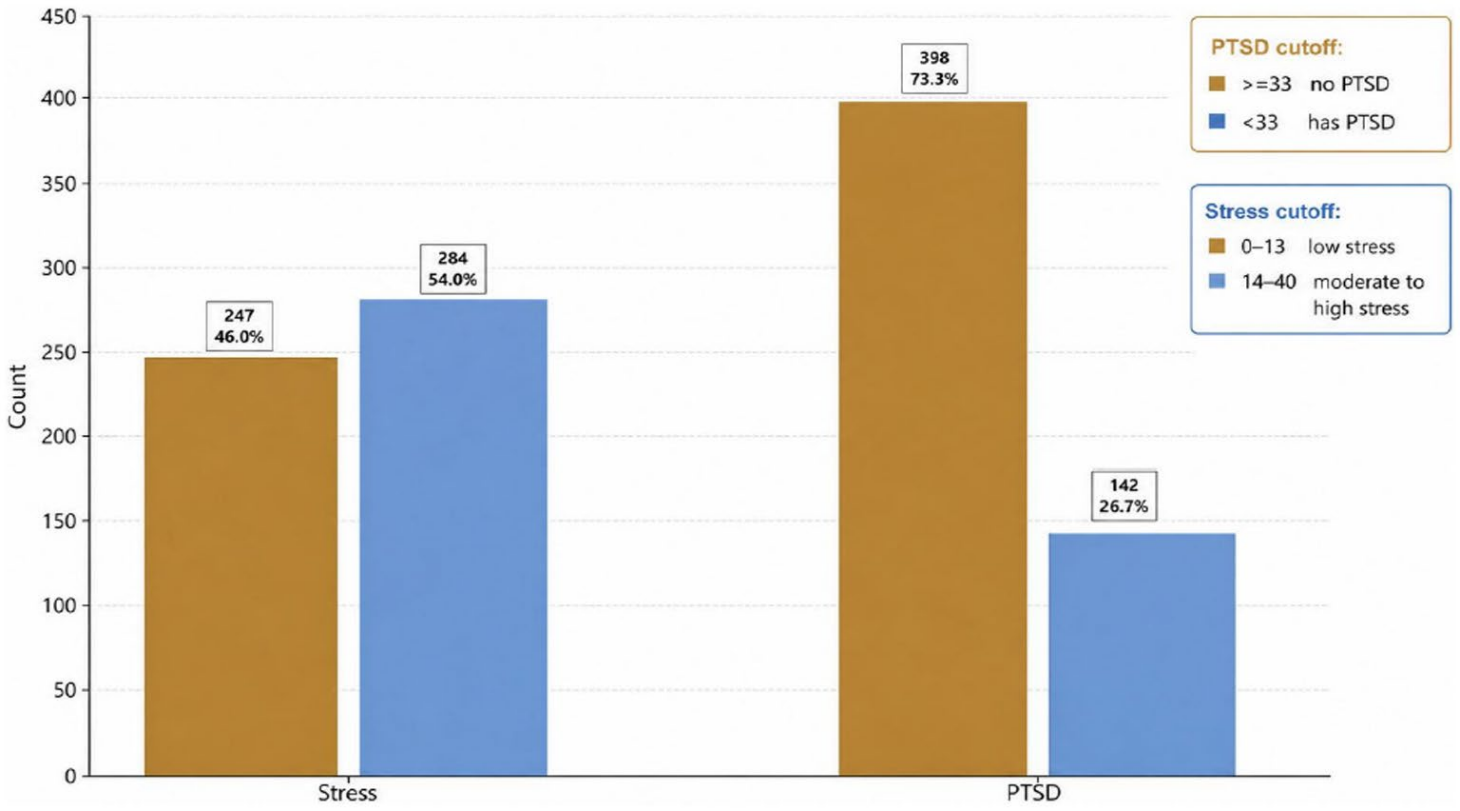
Prevalence of PTSD and perceived stress.

The findings shown in Figure 1 reveal that 26.7% of participants were at risk for PTSD symptoms, while 54% were at risk for moderate-to-high stress levels.

### 3.3 The association between glycemic control and all study variables

The results presented in Table 1 indicate significant associations between glycemic control level (HbA1c) and various factors: marital status (*p* < 0.001), place of residence (*p* < 0.035), PTSD (*p* < 0.001), perceived stress (*p* < 0.001), and dietary pattern (p < 0.001).

### 3.4 Binary logistic regression of poor glycemic control

Binary logistic regression analysis was conducted to identify the factors that were associated to poor glycemic control, as presented in Table 2. The results indicated that individuals with type 2 diabetes were twice as likely to experience poor glycemic control compared to those with type 1 diabetes (AOR: 2.348, *p* < 0.015). Furthermore, participants with PTSD were five times more likely to demonstrate poor control than those without PTSD (AOR: 5.455, *p* < 0.001). Participants experiencing moderate to high levels of stress were three times at a risk to experience poor glycemic control compared to those with low stress levels (AOR: 3.172, *p* < 0.001). Lastly, individuals following average dietary pattern were at low risk to have poor glycemic control than those with a poor dietary pattern (AOR: 0.054, *p* < 0.001).

**Table 2:** Binary logistic regression of poor glycemic control in adults with type 1 and type 2 diabetes mellitus from Southern Governorates of Palestine seeking care at primary healthcare centers in October–November 2025 (*N* = 531).

Characteristics		Poor glycemic control		Adjusted analysis 95% CI, AOR	
		Sig.	AOR	Lower	Upper
Marital status	Others (divorced, widowed, separated)	0.362	1.386	0.687	2.799
	Married	0.067	0.547	0.287	1.043
	Single	Reference			
Type of diabetes	Type 2	0.015	2.348	1.180	4.672
	Type 1	Reference			
PTSD	>33	<0.001	5.455	2.204	13.501
	≤33	Reference			
Stress	Moderate to high	<0.001	3.172	1.867	5.390
	Low	Reference			
Dietary pattern	Average	<0.001	0.054	0.020	0.141
	Good	0.534	0.854	0.520	1.403
	Poor	Reference			

The logistic regression model was adjusted for study variables controlling for sociodemographic variables (age, gender, marital status, governorates, place of residence, work status, current living status, and family monthly income). AOR, adjusted odds ratio; CI, confidence interval. ∗*p* < 0.05. PTSD severity was measured using the PTSD Checklist (PCL-5); a score of >33 indicates a high risk of having PTSD symptoms.

## 4 Discussion

Glycemic control is important as a preventive strategy for individuals at high risk of diabetes and as a therapeutic approach for those with inadequate glycemic management. The current study found that 76.3% of subjects had HbA1c values of 7 or above, indicating a significant prevalence of poor glycemic control which is similar to the findings of other studies (56, 57). Our findings surpass those of previous studies conducted in Iran (66.9%) (68), Sub-Saharan Africa (70%) (69), and Ethiopia (66.8%) (70). Another study indicated that the overall prevalence of poor glycemic control among diabetics in low- and middle-income countries was 69.06% (71). Additionally, a meta-analysis in the Middle East and North Africa found that 63% of individuals with type 2 diabetes (T2D) had poor glycemic control (10). Boye et al. (72) noted that maintaining HbA1c levels below 7% over five-years was linked to a reduced risk of developing diabetes-related complications, such as cardiovascular disease, metabolic disorders, neuropathy, nephropathy, and peripheral vascular disease.

One possible explanation for our finding is the unique environmental conditions and the ongoing and long-lasting political conflict in which these participants live. People living with diabetes are tasked with the ongoing management of a chronic illness, a state that is heavily reliant on regular access to medication and a stable environment to manage their condition daily (73). The constant vigilance required for diabetes management, including monitoring and blood glucose management, is often complicated by the stress of high-conflict environments (73). Securing medications, monitoring and blood glucose management and accessing consistent healthcare become formidable tasks in war-conflict regions, potentially leading to detrimental impacts on the management of diabetes (16). According to a systematic review by Ospelt et al. individuals with diabetes living in war and conflict zones may experience prolonged glucose levels that exceed the recommended range, potentially leading to long-term complications. They face various stressors and factors that negatively impact their quality of life and management of diabetes. Furthermore, they encounter significant disruptions in accessing healthcare, including medications and services (16). In Palestine, economic constraints and irregular family income create financial obstacles that may hinder patients’ ability to afford medications and adhere to their treatment plans. Researchers have identified that limited access to medications and interruptions in medication schedules stem from financial barriers and a lack of knowledge about where to purchase medications (74). Therefore, there is a need to increase the awareness and knowledge of people with diabetes mellitus about the implications of poor glycemic control and the importance of optimizing their treatment plans.

Our research findings identified factors that were significantly associated with poor glycemic control among Palestinians with diabetes mellitus. The findings indicated that participants with type 2 diabetes mellitus were twice as likely to experience poor glycemic control compared to those with type 1 diabetes mellitus. Research suggests that type 2 diabetes is more prevalent among low- to middle-income populations who have limited access to continuous glucose monitoring devices and qualified diabetes specialists (75). Furthermore, in type 1 diabetes mellitus (T1DM), the main issue is the absence of insulin; once insulin is administered, the body usually functions in a predictable manner. In contrast, the primary concern in type 2 diabetes mellitus is insulin resistance, which is characterized by a reduced responsiveness of insulin-targeted tissues to elevated physiological insulin levels and is considered a pathogenic factor in type 2 diabetes (76). Despite the administration of elevated exogenous insulin dosages, cellular glucose uptake in muscle, liver, and adipose tissue remains impaired, complicating glycemic control (75). Additionally, prior research has identified suboptimal adherence as a significant factor associated with poor glycemic control (70, 77–79). To date, no study has directly compared medication adherence between patients with Type 1 Diabetes Mellitus (T1DM) and Type 2 Diabetes Mellitus (T2DM) within the same population in Palestine. Future research should examine adherence patterns across these diabetes groups in the Palestinian context. One study in Palestine found that 42.1% of individuals with type 2 diabetes had low adherence to their treatment and they were not satisfied with their treatment regimens (79). Tegegne et al. revealed that individuals with type 2 diabetes who have limited adherence are 3.67 times more likely to experience suboptimal glycemic control (80). Consequently, Bezie et al. advocated for a greater emphasis on counseling and enhancing adherence, rather than merely modifying medications or adjusting dosages (81). Our findings suggest that individuals with type 2 diabetes may be classified as high-risk individuals due to their increased likelihood of poor glycemic control.

Moreover, the current study found that participants with PTSD had a fivefold higher odds ratio for poor glycemic control compared to those without PTSD. Research indicates that individuals with PTSD are more likely to struggle with blood sugar management (82, 83). Lunkenheimer et al. demonstrated that comorbid PTSD correlates with poorer diabetes-related outcomes, such as diabetic ketoacidosis (DKA) and elevated HbA1c levels, as well as an increased rate of hospital admissions among individuals aged 25 years or younger with T1D, compared to those with T1D alone or those with T1D and another comorbid mental disorder (43). PTSD has been linked to disruptions within the hypothalamic–pituitary–adrenal axis, which is associated with visceral adiposity, insulin resistance, and type 2 diabetes (38, 84). Research by van Bastelaar et al. reported that the co-occurrence of mental health disorders in adults with diabetes (both T1D and T2D) correlates with diminished treatment adherence, suboptimal glycemic control, a higher incidence of emergency department visits for diabetic ketoacidosis, and more frequent hospitalizations (45). Consequently, screening for symptoms of post-traumatic stress disorder is needed for individuals with diabetes mellitus, particularly in areas affected by war and political conflict, where exposure to traumatic events is significantly elevated.

Additionally, the findings of the present study indicated that participants experiencing moderate to high stress were three times at risk of having poor glycemic control compared to those with low stress levels, aligning with previous research (26, 85) and in contradiction to others (86, 87). There is a bidirectional relationship between stress and diabetes, where chronic stress increases the risk of developing diabetes and adversely affects the medical outcomes of individuals with the condition (26). Research shows that emotional stress exposure leads to elevated peripheral glucose levels in people with diabetes (88, 89). The connection between stress and diabetes mellitus may stem from the effects of stress on hormone secretion, as stress triggers the release of hormones like cortisol, which in turn prompts the liver to release glucose, ultimately resulting in higher glycated hemoglobin (HbA1c) levels (90). Elevated levels of distress are associated with poor glycemic control, an increased risk of complications, and lower adherence to treatment regimens (91). Additionally, psychological distress can lead to a decline in quality of life, affecting emotional well-being, social relationships, and daily functioning (92). On the other hand, a diagnosis of diabetes mellitus is a recognized source of considerable psychological distress. Managing diabetes involves complex daily self-care responsibilities, such as blood glucose monitoring, nutritional constraints, medication compliance, and fear over long-term complications, all of which elevate stress in patients with diabetes (93, 94). Consequently, educational programs and standard care services that include stress management strategies should be considered routine therapeutic interventions. Commonly used stress reduction techniques may involve getting sufficient sleep, focusing on positive thoughts before sleeping, and practicing daily relaxation after activities (95).

Finally, the findings of the present study revealed that participants adhering to an average dietary pattern were more likely to have good glycemic control compared to those with a poor dietary pattern, consistent with other research (96–98). This finding aligns with the Nordic Nutrition Recommendations (NNR), which emphasize a balanced diet rich in whole grains, vegetables, fruits, fish, and low-fat dairy products while limiting processed foods, added sugars, and saturated fats. For individuals with DM, this dietary pattern is associated with improved glycemic regulation, lower fasting plasma glucose levels, and better control of HbA1c levels (99–102). The Healthy Eating Index, which is rooted in the National Nutrient Reserve (NNR), assesses the overall quality of the diet. Greater adherence to its recommendations correlates with increased intake of dietary fiber, essential vitamins, and minerals, all of which contribute to blood sugar regulation and the reduction of plasma triglyceride levels (96–98). These findings underscore the importance of promoting NNR-aligned dietary practices as a practical, evidence-based strategy for improving glycemic outcomes in individuals with diabetes mellitus. Contrary to expectations, the good dietary pattern group did not show significantly lower odds of poor glycemic control compared to the poor dietary pattern group. Research indicates that lifestyle modifications focusing on self-monitoring, physical exercise, weight reduction, and diet can significantly enhance glucose control and overall quality of life (103, 104). However, individuals with a “good” dietary pattern may still engage in unhealthy lifestyle behaviors, such as poor medication adherence or physical inactivity, which can diminish the potential glycemic benefits of their diet (105). Furthermore, the ability to detect a significant difference may have been constrained by the small number of participants within certain subgroups (106). Therefore, additional research is required to explore the reasons behind the absence of association between poor glycemic control and a good dietary pattern.

Nevertheless, despite the significant findings of the current study—being the first in Palestine to target both types of diabetes mellitus and their association with PTSD—our research had some limitations. The use of a cross-sectional design restricts the ability to establish causal relationships between the research variables and raises the potential for reverse causality. The dependence on self-reported data constitutes another possible limitation, as it may include biases such as social desirability or recall bias. Social desirability bias in self-reports may lead participants to inaccurately report their consumption of “healthy” foods, PTSD symptoms, or stress levels. Additionally, recall bias in the Food Frequency Questionnaire (FFQ) may result in measurement errors that significantly misclassify food intake, particularly among patients with psychosocial comorbidities who may struggle with retrospective recall. Future research in this population should incorporate FFQ data alongside at least two repeated 24-h dietary recalls. Furthermore, the inclusion of two governorates (Bethlehem and Hebron) may restrict the generalizability of the study to other individuals with diabetes mellitus in different governorates. Additional limitations include sampling bias, as the study only includes individuals who utilized primary healthcare facilities. It also does not evaluate all factors that may influence glycemic control, such as medication adherence, physical activity, specific trauma types, food insecurity, and severity. Finally, in this study, associations are presented using odds ratios. It is important to note that in cross-sectional studies, the odds ratio (OR) may exaggerate the prevalence ratio, particularly when the outcome of interest is common. Future research could benefit from employing generalized linear models to provide direct estimates of prevalence ratios. Future studies may address these limitations by utilizing a longitudinal design to more effectively evaluate causal associations across time.

### 4.1 Implications for practice

Our research findings identified factors that are associated with poor glycemic control in persons with diabetes mellitus, which may have significant implications for disease progression. The results of this study could assist policymakers and healthcare practitioners in developing tailored protocols aimed at enhancing diabetes management and glycemic control within primary healthcare settings. Longitudinal studies are needed to investigate the causal relationship and the factors affecting glycemic control among persons with diabetes and to evaluate causal relationships. Moreover, further longitudinal research could explore additional variables such as depression, anxiety, medication adherence, physical activity, specific trauma types, food insecurity and severity and the impact of political factors and its association with poor glycemic control.

## 5 Conclusion

The findings of this study revealed a significant prevalence of poor glycemic control, especially among participants with PTSD, psychological distress, type 2 diabetes, and unhealthy dietary patterns. Consequently, it may be beneficial that psychological interventions and dietary counseling be made available at primary health care centers to support people with diabetes mellitus in managing their blood sugar levels, preventing complications, and improving their quality of life.

## Data Availability

The original contributions presented in the study are included in the article/supplementary material, further inquiries can be directed to the corresponding author/s.
